# Assessment of Patient-Reported Outcomes at 48 Months of Treatment with Dupilumab for Severe Atopic Dermatitis: A Single-Center Real-Life Experience with 126 Patients

**DOI:** 10.3390/ph17010117

**Published:** 2024-01-16

**Authors:** Francesca Barei, Martina Zussino, Simona Tavecchio, Luisa Angileri, Arianna Rizzo, Paolo Calzari, Angelo V. Marzano, Silvia Ferrucci

**Affiliations:** 1 Dermatology Unit, Fondazione IRCCS Ca’ Granda Ospedale Maggiore Policlinico, 20122 Milan, Italy; 2 Department of Pathophysiology and Transplantation, Università degli Studi di Milano, 20122 Milan, Italy

**Keywords:** atopic dermatitis, dupilumab, patient-reported outcomes

## Abstract

Background: The main objective was to analyze patient-reported outcomes (PRO) trends over a four-year period in severe atopic dermatitis (AD) patients treated with dupilumab. Methods: data from 126 severe patients receiving dupilumab for at least 48 months were collected. The clinical scores assessed included the Eczema Area and Severity Index (EASI), Pruritus Numerical Rating Scale (NRS), Sleep NRS, Patient-Oriented Eczema Measure (POEM), Dermatology Life Quality Index (DLQI), and Atopic Dermatitis Control Tool (ADCT). Results: the study compellingly demonstrates dupilumab’s effectiveness in reducing EASI and improving PROs, with sustained enhancements observed beyond the initial twelve months of treatment. Univariate and multivariate regression analyses show that baseline factors do not significantly increase the risk of adverse outcomes related to Pruritus NRS, POEM, or ADCT at T48. The robust correlation between ADCT and other PROs suggests closely aligned changes. Conclusion: Dupilumab’s benefits endure beyond the first year, emphasizing its long-term efficacy, and consistently improves AD outcomes regardless of individual characteristics or clinical variables. ADCT appears to be a practical and versatile tool for the streamlined assessment of AD treatment outcomes.

## 1. Introduction

Atopic dermatitis (AD) is a chronic, inflammatory skin disease, characterized by intense itch and recurrent eczematous skin lesions. Common features seen in most patients with AD are generalized skin dryness, early disease onset (typically in the first 2 years of life), and a personal history or family history, or both, of atopic disease (e.g., asthma, allergic rhinitis, and atopic dermatitis) or specific immunoglobulin E reactivity [1]. AD is one of the most common chronic diseases worldwide, with a global annual prevalence of 3.5% (230 million). AD can manifest at any point in life, but the incidence peaks in infancy, with onset before 6 years of age in an estimated 80% of patients. According to a meta-analysis of seven birth-cohort studies with follow-up times of up to 26 years, the annual prevalence in adult patients is up to 14.6% in developed countries, making AD a lifelong disease [2,3].

Regarding its symptoms and associated comorbidities, AD is linked to sleep disturbance, diminished quality of life, anxiety, and depression. Itching stands out as a cardinal symptom of AD and plays a pivotal role in determining patients’ psychosocial well-being and quality of life. The intensity of the itch correlates with a decline in quality of life and the presence of depression symptoms [4]. Another prevalent symptom of AD is sleep disturbance, often a consequence of itching. Eczema is associated with a heightened risk of fatigue, regular daytime sleepiness, and recurrent insomnia, all of which are potential predictors of a poorer overall health status [5]. AD places a significant burden on patients, stemming from the impact of symptoms and visible physical manifestations of the disease. This burden extends to various aspects, negatively affecting the quality of life (QoL), sleep, self-esteem, interpersonal relationships, and participation in leisure and sports, as well as attendance or performance at school or work [6]. Furthermore, as AD ranks among the most common diseases in developed countries, the associated costs should not be overlooked. The financial impact of AD encompasses direct costs for patients and families (e.g., prescriptions, outpatient visits, and hospitalization) as well as indirect costs for patients and society, including work and school absenteeism, reduced productivity while at work or school (presenteeism), and potential costs linked to decreased quality of life [7].

Interleukin (IL)-13 and IL-4 are key drivers of underlying inflammation in AD, leading to skin-barrier dysfunction, immune dysregulation, and, ultimately, chronic type-2 inflammation [1,2,3]. Dupilumab is a humanized IgG4 monoclonal antibody that targets the IL-4 receptor alpha chain (IL-4Rα), common to both IL-4R complexes, type 1 (IL-4 specific) and type 2 (IL-4 and IL-13 specific), thus blocking both IL-4 and IL-13 signaling [8,9].

The Eczema Activity and Severity Index (EASI) is used as the primary measure to assess clinical response in AD studies. But, it is assessed by clinicians, and it may often not adequately reflect patients’ experiences with the disease. On the contrary, patient-reported outcomes (PROs) assess the patient-reported symptoms and their impacts on the patient’s quality of life. PROs provide a valuable window into the patient’s perspective, shedding light on the physical, emotional, and social aspects of the disease [10]. By examining these trends over an extended timeframe, it provides insights into the long-term efficacy of a treatment. Several validated PRO measures can be used to assess different clinical aspects, such as itch intensity (Pruritus Numerical Rating Scale), sleep (Sleep Numerical Rating Scale), quality of life (Dermatology Life Quality Index), patient’s perception of the disease (Atopic Dermatitis Control Tool and the Patient-Oriented Eczema Measure), and anxiety and depression (Hospital Anxiety and Depression Scale).

The SOLO 1 and SOLO 2 trials demonstrate the efficacy of dupilumab in alleviating atopic dermatitis symptoms, with a 4.5% and 4.0% mean reduction in Peak Pruritus NRS scores by day 2. Patients on dupilumab also experienced significant improvement in POEM scores by week 2, indicating rapid relief from symptoms. Quality of life, as measured by DLQI, showed a positive trend, with more dupilumab-treated patients reporting ‘not at all’ for each DLQI item compared to the placebo [11,12,13]. Moreover, the long-term effectiveness of dupilumab is evident, as patient-reported benefits were maintained during treatment for up to 3 and 4 years according to a RELIEVE-AD study and two open-label extension studies [14,15,16]. To date, there is limited information on patient-reported outcomes (PROs) during real-life treatment with dupilumab. Real-life studies on PROs during long-term dupilumab treatment are scarce, although existing data indicate positive long-term outcomes related to itch, quality of life, and disease control [17,18].

## 2. Results

The baseline epidemiological and clinical characteristics of our population are summarized in Table 1.

As for the 20 patients that discontinued the treatment, 2 (10%) patients discontinued due to conjunctivitis, 4 (20%) due to facial redness, 1 (5%) due to hypereosinophilia, 3 (15%) due to psoriasis, 3 (15%) due to ineffectiveness, 4 (20%) due to loss of efficacy, and 3 (15%) due to the patient’s choice.

### 2.1. Eczema Assessment and Severity Score (EASI)

The median (Q1–Q3) score for EASI was 26.0 (24.0–34.0) at the baseline and rapidly decreased to 7.0 (4.0–11.0) at T1 (*p*-value < 0.001 vs. baseline), 4.0 (1.75–7.0) at T4, 2.0 (1.0–5.0) at T12, and 1.0 (0.0–3.0) at T24. After T20, the trend remained stable, with a median score of 1.0 (0.0–1.0) at T48. The median (Q1–Q3) percentage improvement of EASI from the baseline was 77.2 (61.6–87.5) at T1, 85.7 (75.6–94.7) at T4, 91.0 (83.8–95.8) at T8, and 93.2 (83.3–97.1) at T12. After T12, there was further progressive improvement until T48, where the median improvement reached 97.6 (95.8–100.0).

### 2.2. Pruritus Numerical Rating Scale (Pruritus NRS) and Sleep Numerical Rating Scale (Sleep NRS)

**Figure 1:**
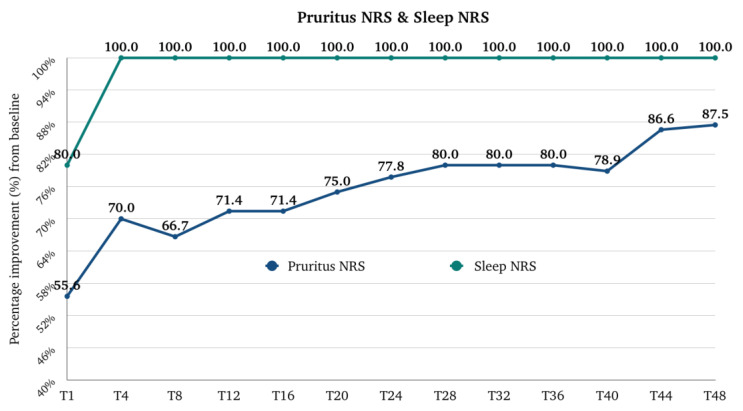
Percentage improvement of Pruritus NRS (Numerical Rating Scale) and Sleep NRS from baseline after one month of treatment (T1), after 4 months of treatment (T4), then every 4 months till 48 months (T48) of treatment. NRS, Numerical Rating Scale; T, time point in months.

**Figure 2:**
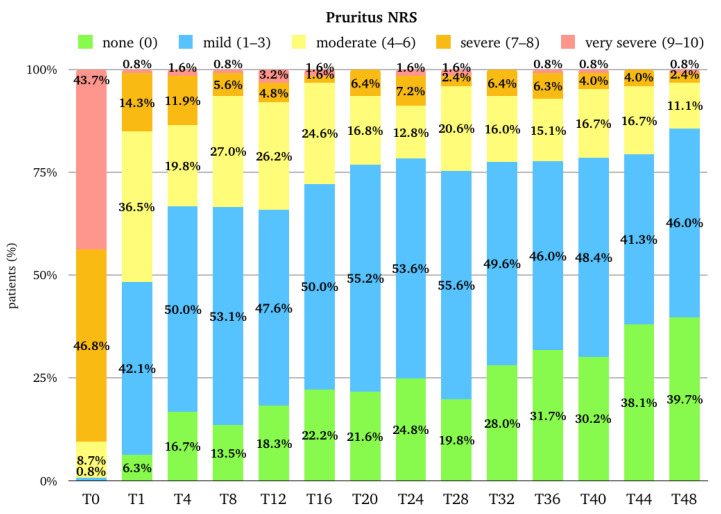
Distribution of the population (expressed in percentage) in the different Pruritus NRS (Numerical Rating Scale) categories at baseline (T0), after one month of treatment (T1), after 4 months of treatment (T4), and then every 4 months till 48 months (T48) of treatment. T, time point in months; NRS, numerical rating scale.

The median (Q1–Q3) score for Pruritus NRS was 8.0 (7.0–10.0) at the baseline, and it rapidly decreased to 4.0 (2.0–5.0) at T1 (*p*-value < 0.001 vs. baseline), to 2.5 (1.0–4.0) at T4, then to 2.0 (1.0–4.0) at T12. After T12, the trend remained stable, with a median score of 2.0 (0.0–3.0) at T40. There is a further improvement at T48, as the median is 1.0 (0.0–2.3). The median (Q1–Q3) percentage improvement of NRS Pruritus from the baseline was 55.6 (33.3–75.0) at T1, 70.0 (44.4–85.7) at T4, and 71.4 (42.9–89.1) at T12. After T12, there is a further progressive improvement until T48, where the median improvement is 87.5 (66.7–100.0) (Figure 1). The percentage of patients with a very severe itch (NRS 9–10) and a severe itch (NRS 7-8) rapidly decreased from 43.7% and 46.8% at the baseline to 0.8% and 14.3% at T1. At T12, 3.2% and 4.8%, respectively, belonged to these two groups. At T48, only 0.8% and 2.4% of patients had a very severe itch or a severe itch, respectively (Figure 2).

The median (Q1–Q3) score for Sleep NRS was 7.0 (5.0–9.0) at the baseline, and it rapidly decreased to 0.0 (0.0–3.0) at T1 (*p*-value < 0.001 vs. baseline). The trend remained stable, with a median score of 0.0 (0.0–0.0) at T48. The median (Q1–Q3) percentage improvement of Sleep NRS from the baseline was 80.0 (50.0–100.0) at T1 and 100.0 (80.0–100.0) at T4 (Figure 1).

### 2.3. Patient-Oriented Eczema Measure (POEM)

**Figure 3:**
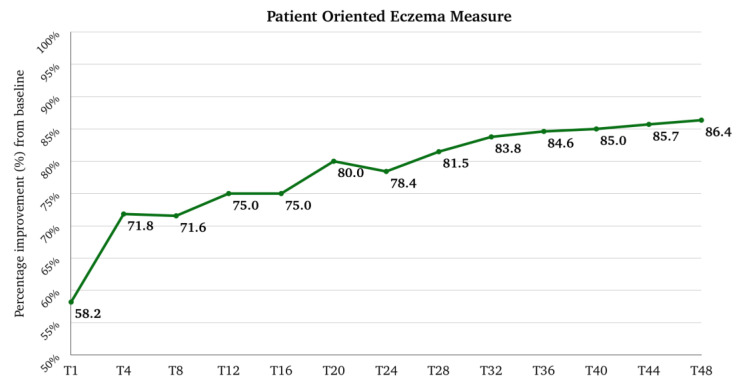
Percentage improvement of patient-oriented eczema measured from baseline after one month of treatment (T1), after 4 months of treatment (T4), and then every 4 months till 48 months (T48) of treatment. T, time point in months.

**Figure 4:**
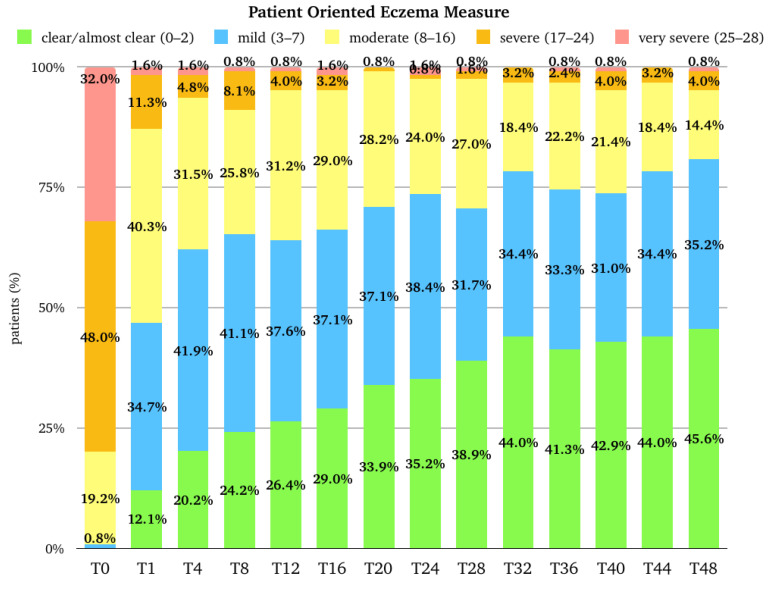
Distribution of the population (expressed in percentage) in the different Patient-Oriented Eczema Measure categories at baseline (T0), after one month of treatment (T1), after 4 months of treatment (T4), and then every 4 months till 48 months (T48) of treatment. T, time point in months.

The median (Q1–Q3) score for POEM was 22.0 (17.0–26.0) at the baseline, and it rapidly decreased to 8.0 (5.0–12.8) at T1 (*p*-value < 0.001 vs. baseline), 5.0 (3.0–10.0) at T4, 5.0 (2.0–10.0) at T12, and 4.0 (2.0–9.0) at T16. After T16, the trend remained stable until T28; at T32, the median score was 3.0 (1.0–7.0), which was maintained until T48. The median (Q1–Q3) percentage improvement of POEM from the baseline was 58.2 (35.3–76.5) at T1, 71.8 (47.2–84.9) at T4, and 75.0 (53.6–88.2) at T12. After T12, there was further progressive improvement until T48, where the median improvement reached 86.4 (68.5–95.8) (Figure 3). The percentage of patients with a very severe POEM (25–28) and a severe POEM (17–24) rapidly decreased from 32.0% and 48.0% at the baseline to 1.6% and 11.3% at T1. At T12, 0.8% and 4.0%, respectively, belonged to these two groups. The results at T48 were similar (Figure 4).

### 2.4. Atopic Dermatitis Control Tool (ADCT)

**Figure 5:**
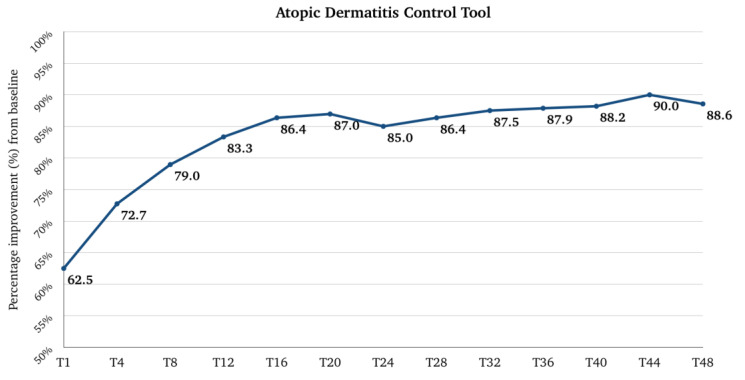
Percentage improvement of Atopic Dermatitis Control Tool from baseline after one month of treatment (T1), after 4 months of treatment (T4), and then every 4 months till 48 months (T48) of treatment. T, time point in months.

**Figure 6:**
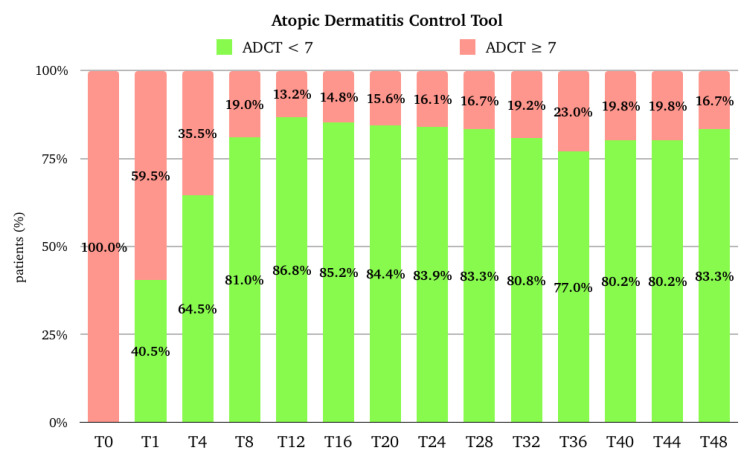
Distribution of the population (expressed in percentage) in the different Atopic Dermatitis Control Tool categories at baseline (T0), after one month of treatment (T1), after 4 months of treatment (T4), and then every 4 months till 48 months (T48) of treatment. ADCT, Atopic Dermatitis Control Tool; T, time point in months.

The median (Q1–Q3) score for ADCT was 21.0 (17.8–23.0) at the baseline, and it rapidly decreased to 7.0 (6.0–9.0) at T1 (*p*-value < 0.001 vs. baseline), 5.0 (3.5–7.0) at T4, and 3.0 (2.0–5.0) at T12. After T12, the trend remained stable until T28; at T32, the median score was 2.0 (0.0-5.5), which was maintained until T48. The median (Q1–Q3) percentage improvement of ADCT from the baseline was 62.5 (52.8–71.4) at T1, 72.7 (64.7–82.6) at T4, and 83.3 (75.0–91.3) at T12. After T12, there was a further slight, progressive improvement until T48, where the median improvement reached 88.6 (73.2–100.0) (Figure 5). The percentage of patients with an ADCT score ≥ 7 (AD “not in control”) rapidly decreased from 100% at the baseline to 59.5% at T1. At T12, 13.2% of patients had a score ≥ 7. At T48, the percentage is slightly higher (16.7%) (Figure 6).

### 2.5. Dermatology Life Quality Index (DLQI)

**Figure 7:**
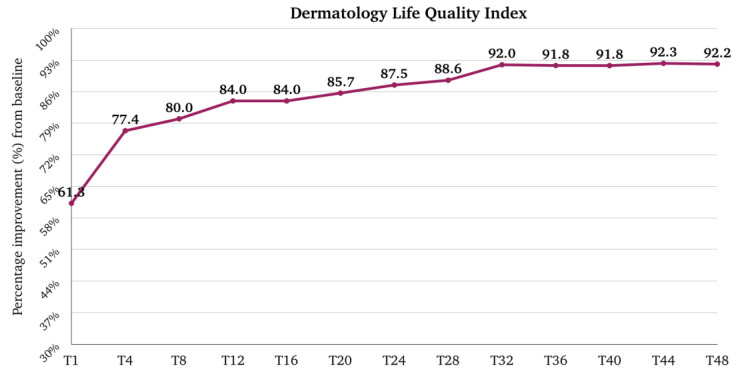
Percentage improvement of Dermatology Life Quality Index from baseline after one month of treatment (T1), after 4 months of treatment (T4), and then every 4 months till 48 months (T48) of treatment. T, time point in months.

**Figure 8:**
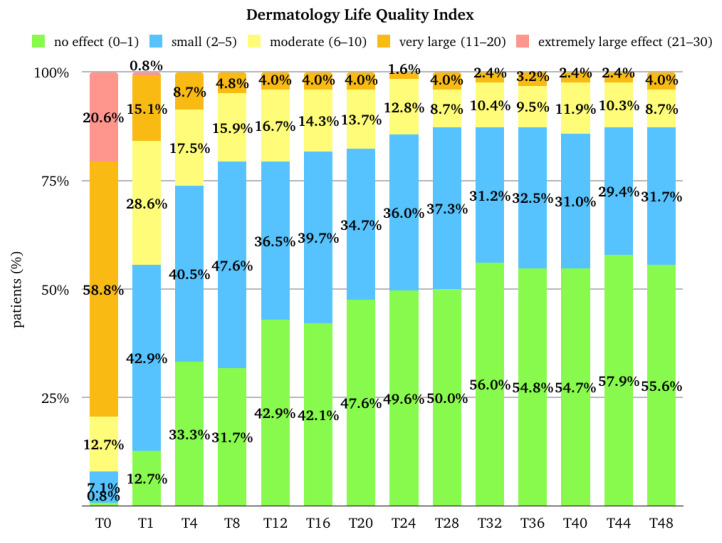
Distribution of the population (expressed in percentage) in the different Dermatology Life Quality Index categories at baseline (T0), after one month of treatment (T1), after 4 months of treatment (T4), and then every 4 months till 48 months (T48) of treatment. T, time point in months.

The median (Q1–Q3) score for DLQI was 15.0 (11.0–19.0) at the baseline, and it rapidly decreased to 5.0 (2.0–9.0) at T1 (*p*-value < 0.001 vs. baseline), 3.0 (1.0–6.0) at T4, and 2.0 (1.0–4.3) at T12. After T12, the trend remained stable until T28; at T32, the median score was 1.0 (0.0–3.0), which was maintained until T48. The median (Q1–Q3) percentage improvement of DLQI from the baseline was 61.3 (39.6–84.6) at T1, 77.4 (58.3–90.9) at T4, and 84.0 (64.7–94.4) at T12. After T12, there was further progressive improvement until T48, where the median improvement reached 92.2 (76.4–100.0) (Figure 7). The percentage of patients with an extremely large effect (21–30 points) and a very large effect (11–20) on the quality of life rapidly decreased from 20.6% and 58.0% at the baseline to 0.8% and 15.1% at T1. At T12, 0.0% and 4.0%, respectively, belonged to these two groups. The results at T48 were similar (Figure 8).

### 2.6. Correlation Analysis

Spearman’s rank correlation coefficient (ρs) was used to assess the potential correlation between the scores. The results showed that the POEM and ADCT scores, except for T0, are correlated at each time point (*p*-value < 0.001) and exhibit a strong correlation (ρs ≥ 0.7) after T28. Additionally, the results revealed that the DLQI and ADCT scores are correlated at each time point (*p*-value < 0.001) and exhibit a strong correlation after T24. Furthermore, the results indicated that the Pruritus NRS and ADCT scores are correlated at each time point (*p*-value < 0.001), except for T0, and exhibit a strong correlation after T24 (Table S1: correlation analysis between ADCT and POEM, ADCT and DLQI, and ADCT and Pruritus NRS at each time point).

### 2.7. Regression Analysis

We performed univariate regression and multivariate regression analyses to evaluate a potential predominant factor affecting the determination of moderate or severe or very severe Pruritus NRS (score ≥ 4 points), a moderate or severe or very severe POEM score (score ≥ 8), and a score of ≥7 for ADCT at T48. As covariates, we considered sex, baseline age, intrinsic pattern vs. extrinsic pattern, AD onset pattern, atopic family history, number of atopic comorbidities, AD phenotype, and sensitization to a contact allergen. No collinearity was found between these variables. In the univariate regression and the multivariate regression analyses, no factor among the ones cited above appeared to increase the risk of having an NRS Pruritus score ≥ 4, a POEM score ≥ 8, and a score of ≥7 for ADCT at T48 (Table S2: univariate regression and multivariate regression analyses for Numerical Rating Scale Pruritus; Table S3: Univariate regression and multivariate regression analyses for POEM; Table S4: Univariate regression and multivariate regression analyses for ADCT).

### 2.8. Safety and Tolerability

Among the 126 individuals included in the analysis, 18.2% reported eosinophilia (grade I), 21.4% reported conjunctivitis (grade I or II), 2.4% reported asthenia (grade I), 0.8% reported a herpetic infection (grade II), 0.8% reported the concomitant onset of psoriasis (grade II), and 16.7% reported dupilumab-induced facial redness (grade II). No serious side effects were documented. Adverse events were graded according to the common toxicity criteria for adverse events (CTCAE). 

## 3. Discussion

### 3.1. Descriptive Analysis

In conclusion, the study provides compelling evidence for the effectiveness of dupilumab in the treatment of atopic dermatitis (AD), not only in reducing clinical scores but also in improving patient-reported outcomes (PROs), including symptom severity, itch, sleep quality, disease control, and overall quality of life. These findings are indicative of the substantial and sustained benefits of dupilumab therapy for patients with AD. Moreover, the study findings indicate that the improvement in patients’ conditions continues beyond the initial twelve months of treatment (T12). This progressive improvement underscores the sustained and ongoing benefits of dupilumab therapy for patients with AD.

Drawing comparisons with results from clinical trials, in contrast to the findings in the RELIEVE-AD study at the 3-year mark [14], the DLQI score in this study is lower than reported by the authors, though the proportion of patients reporting a DLQI score of 0–1 is similar. Concerning the ADCT results, the percentage improvement at 3 years was lower compared to those in the RELIEVE-AD study (75.9% vs. 87.9%), but the percentage of patients with controlled AD was higher in the RELIEVE-AD study compared to the present study (80.7% vs. 77.0%). Furthermore, in comparison to outcomes observed in a four-year open-label study [15], the results for Pruritus NRS are comparable after one year of treatment and slightly superior after four years (1.0 vs. 2.10).

Regarding real-life studies, in comparison to the findings of Ortoncelli et al. [17], Pruritus and Sleep NRS scores in this study demonstrate superiority at T4, T12, and T24, although it is important to note that the authors had a higher baseline Pruritus NRS. However, at T36 (3 years of treatment), the scores become comparable. Ortoncelli et al.’s DLQI scores consistently exhibit a slight elevation (an average difference of 1.5) at every time point compared to the present study. Nevertheless, the percentage of patients achieving a DLQI score of 0/1 at T36 is higher in their study (61.7% vs. 54.8%) than in this study. Similar trends are observed for POEM, though the authors had a higher baseline score. Notably, Ortoncelli et al. did not investigate ADCT. Additionally, the results align with those of Miniotti et al. [18], reflecting a comparable trend in the mean scores of Pruritus NRS, Sleep NRS, POEM, and DLQI. While the POEM and DLQI scores at T36 are slightly lower (almost one point), it is essential to highlight that Miniotti et al. did not consider ADCT in their analysis. In summary, findings from this study are in line with real-life studies at the 3-year mark of treatment, indicating overall comparability in the observed trends and outcomes.

### 3.2. Correlation Analysis

In our correlation analysis, the results indicate a strong and consistent correlation between the ADCT and the POEM, ADCT, and the DLQI and ADCT and the Pruritus NRS after the first month of treatment. Importantly, the sustained strong correlation beyond T28 underscores their reliability for assessing treatment effectiveness over the long term. The strong and consistent correlation between the ADCT and the other PROs indicates that changes in one measure correspond closely with changes in the others. This suggests that improvements or deteriorations in ADCT scores are reflective of similar changes in other important indicators of atopic dermatitis. ADCT presents as a practical alternative for assessing treatment outcomes. This could streamline clinical assessments, making ADCT a versatile and efficient tool for evaluating AD symptom control. Together with the EASI, ADCT can give a good clinical overall assessment of a patient during the follow up.

### 3.3. Regression Analyses

Regarding the multivariate regression analyses, our results suggest that the severity of atopic dermatitis in patients, as measured by Pruritus NRS, POEM, and ADCT, does not significantly vary based on the demographic or clinical characteristics explored in this study. Overall, these findings emphasize the robustness and consistency of the therapeutic effects of the treatment, likely related to the use of dupilumab, in the management of AD. The treatment appears to be effective in addressing itching symptoms, overall disease severity, and disease control for a wide range of AD patients, regardless of demographic characteristics, disease history, or clinical factors. This underscores the universal benefits of dupilumab for the management of AD and provides reassurance to both clinicians and patients that the treatment can offer consistent improvements in AD outcomes, regardless of individual patient characteristics or clinical variables. While these findings provide a significant contribution to understanding the effectiveness of treatment in AD management, further research may explore additional factors or patient-specific variations that could play a role in shaping individual responses to therapy.

### 3.4. Strengths and Limitations

The key strengths of this study encompass its substantial sample size and the extended follow-up duration. To our knowledge, this represents the initial real-life study documenting patient-reported outcome (PRO) trends over a 4-year treatment period. Additionally, acknowledging the robust and consistent correlation between ADCT and other PROs, we emphasize the potential of ADCT as a user-friendly and versatile tool, suggesting its use as a daily replacement for the other PROs. Furthermore, the regression analyses underscore the efficacy of dupilumab irrespective of individual patient characteristics or clinical variables. This study stands as the sole real-life investigation that scrutinized clinical or epidemiologic characteristics as potential predominant factors influencing the determination of a moderate or severe score after 4 years of treatment.

The study’s main limitation is its exclusive focus on a subgroup of 126 “best-responders” who stayed on a four-year therapy, omitting those who had to stop due to adverse effects or inadequate response. Consequently, the findings are informative for this specific group but may not fully represent the real-world diversity seen in clinical practice. Moreover, dupilumab cannot currently be compared with another biologic or small-molecule drug, since it is the only biologic drug for atopic dermatitis for which we may have available, at least in real life, data at 4 years of therapy.

## 4. Materials and Methods

### 4.1. Population

The study population consists of patients with atopic dermatitis under treatment with dupilumab, who are affiliated with the Allergological Dermatology Service at the Dermatology Unit of the Fondazione IRCCS Ca’ Granda Ospedale Maggiore Policlinico of Milan (Italy). The study was conducted solely at the aforementioned center in Milan (single-center study). This is a retrospective study. Ethical approval is referred to as protocol Dupi Long Term 2022. The patients in this manuscript have given written informed consent to the publication of their case details. 

Among the patients who started dupilumab treatment before October 2019 (*n* = 160), 6 patients were lost due to city moving, 2 patients were lost since they no longer showed up for the follow-up, 21 discontinued the therapy, 3 are currently under temporary suspension (due to pregnancy), 2 suspended the drug temporarily, and 126 are actively receiving treatment. We collected those 126 patients with severe atopic dermatitis who were treated with dupilumab for at least 48 months at standard doses (600 mg at the baseline, then 300 mg every other week). We excluded patients who had discontinued therapy even temporarily for more than one month.

### 4.2. Data Collection

At baseline, we acquired the data of sex, age, height, baseline weight, atopic comorbidities (rhinitis, conjunctivitis, asthma, food allergy nasal polyposis, and eosinophilic esophagitis), other comorbidities, AD phenotype (classified as proposed by Salvador et al. [19]), involvement of specific site (face, hands, or genitals), atopic family history, AD-onset age, AD-onset pattern (early-onset [E.O.] persistent, E.O. relapsing, and late-onset), previous use of systemic drugs for AD, and intake of systemic drug for AD at the baseline. As clinical scores, EASI, and the PROs, Pruritus NRS, sleep NRS, POEM, ADCT, and DLQI were used. EASI was calculated either manually or using an online tool [20]. The itching and sleep scores were directly reported by the patient, while the DLQI, POEM, and ADCT scores were collected through the specific questionnaire validated in various languages. Pruritus NRS was classified into five categories: none (0 points), mild (1–3 points), moderate (4–6 points), severe (7–8), and very severe (9–10) [21]. POEM was classified as follows: 0–2 (clear/almost clear), 3–7 (mild), 8–16 (moderate), 17–24 (severe), and 25–28 (very severe) [22]. ADCT was classified as follows: 0–7 (under control) and ≥7 points (not in control) [23]. DLQI was classified as follows: no effect on the patient’s life (0–1 points), small effect (2–5 points), moderate effect (6–10 points), very large effect (11–20), and extremely large effect (21–30 points) [24]. We considered the data collected at the baseline (T0), after one month of treatment (T1), after 4 months of treatment (T4), and then every 4 months till 48 months (T48) of treatment.

### 4.3. Objectives

This study aims to evaluate the trend of patient-reported outcomes (PROs) during dupilumab treatment and their correlation with clinical response. We present data from the patient population of a single tertiary care center, covering up to 48 months of dupilumab treatment for severe atopic dermatitis. The primary objective of this study is to assess trends in PROs over four years in severe atopic dermatitis patients treated with dupilumab. Additionally, we aim to investigate potential correlations between various PRO measures, examining whether changes in one measure closely correspond with changes in others. Furthermore, we seek to identify a potential predominant factor influencing specific PROs at the 48-month mark of treatment.

### 4.4. Statistical Analysis

Statistical analyses were undertaken using SPSS software (IBM, version 29.0). Descriptive statistics are reported as mean and standard deviation or median and 25–75° quartile for quantitative variables based on the distribution of the population. Absolute numbers and frequencies are used for categorical variables. Either Friedman’s test or an ANOVA for repeated measures was used to compare the scores at different times based on the distribution of the variable. Pearson’s correlation coefficient or Spearman’s rank correlation coefficient, as appropriate, was used to assess a potential correlation between the scores. We performed univariate regression and multivariate regression analyses to evaluate a potential predominant factor affecting the determination of moderate or severe or very severe Pruritus NRS (score ≥ 4 points), a moderate or severe or very severe POEM score (score ≥ 8), and a score of ≥7 for ADCT at T48. As covariates (potential factors), we considered sex (female vs. male), baseline age (over 60 years old versus under 60 years old), intrinsic pattern vs. extrinsic pattern, AD onset pattern (late onset vs. early onset), atopic family history (yes vs. no), atopic comorbidities (3 or more comorbidities vs. 0 comorbidities and 1–2 comorbidities vs. 0 comorbidities), AD phenotype (prurigo nodularis [PN] vs. classical phenotype and phenotypes other than classical and PN vs. classical), and sensitization to a contact allergen (yes vs. no). All statistical analyses were 2 tailed and performed using an alpha error = 0.05. A *p*-value < 0.05 was considered significant.

## 5. Conclusions

In conclusion, the study provides robust evidence of dupilumab’s efficacy in AD management, reducing clinical scores and enhancing PROs, including symptom severity, itch, sleep quality, disease control, and overall quality of life. This improvement extends beyond the initial 12 months of treatment, underlining the sustained benefits of dupilumab for AD patients. Given ADCT’s strong and consistent correlations with various scores, it presents a practical alternative for assessing treatment outcomes. Moreover, our study assures consistent AD outcome improvements irrespective of individual characteristics or clinical variables, although further research may explore other factors affecting individual therapy responses.

## Figures and Tables

**Table 1:** Epidemiological characteristics of our population of 126 patients. AD, atopic dermatitis.

Patients (*n*)	126
Age, mean ± DS	39.89 ± 16.6
Sex, *n* (%)	
- Male	75 (59.5)
- Female	51 (40.5)
Onset age, median (Q1–Q3)	1.0 (0.0–20.0)
AD onset pattern, *n* (%)	
- Early-onset persistent	63 (50.0)
- Early-onset relapsing	25 (19.8)
- Late onset	38 (30.2)
Atopic comorbidities, *n* (%)	
- Rhinitis	97 (77.0)
- Conjunctivitis	76 (60.3)
- Asthma	54 (42.9)
- Eosinophilic esophagitis	1 (0.8)
- Nasal polyposis	1 (2.4)
- Food allergy	27 (21.4)
- Drug allergy	1 (0.8)
AD phenotype, *n* (%)	
- Classical	54 (42.9)
- Generalized inflammatory	27 (21.4)
- Generalized lichenified	19 (15.1)
- Erythrodermic	7 (5.6)
- Prurigo nodularis	13 (10.3)
- Nummular eczema	6 (4.8)
Concomitant contact allergy, *n* (%)	28 (22.2)
Positive family history for atopy, *n* (%)	57 (45.2)
Other comorbidities, *n* (%)	
- Keratoconus	3 (2.4)
- Alopecia areata	2 (1.6)
- Hypertension	17 (13.5)
- Diabetes	5 (4.0)
- Dyslipidemia	5 (4.0)
- Hypothyroidism	4 (3.2)
- Celiac disease	3 (2.4)
Previous systemic treatment, *n* (%)	
- Cyclosporin	108 (85.7)
- Methotrexate	17 (13.5)
- Azathioprine	5 (4.0)
- Abrocitinib	1 (0.8)
- Baricitinib	1 (0.8)
- Tralokinumab	0 (0.0)
- Upadacitinib	0 (0.0)
Involvement of specific site at baseline, *n* (%)	
- Face	115 (91.3)
- Hand	110 (87.3)
- Genitals	51 (40.5)

## Data Availability

Data are unavailable due to privacy restrictions. The data presented in this study are available on request from the corresponding author.

## Supplementary Materials

The following supporting information can be downloaded at: https://www.mdpi.com/article/10.3390/ph17010117/s1. Table S1: correlation analysis between ADCT and POEM, ADCT and DLQI, and ADCT and Pruritus NRS at each time point; Table S2: univariate regression and multivariate regression analyses for Numerical Rating Scale Pruritus; Table S3: Univariate regression and multivariate regression analyses for POEM; Table S4: Univariate regression and multivariate regression analyses for ADCT.
