# Supplementary material for: Assessment of Patient-Reported Outcomes at 48 Months of Treatment with Dupilumab for Severe Atopic Dermatitis: A Single-Center Real-Life Experience with 126 Patients
Source: Pharmaceuticals (Basel). 2024 Jan 16;17(1):117. doi: 10.3390/ph17010117 (PMC10821420; doi:10.3390/ph17010117)
Supplement: Supplementary file 1 [file pharmaceuticals-17-00117-s001.zip › pharmaceuticals-2792307-supplementary.pdf]

Table S1. Correlation analysis between ADCT and POEM, ADCT and DLQI and ADCT and Pruritus NRS at each time point. ρ_s, Spearman's rank correlation coefficient; PROs, patient-reported outcomes; P-NRS, Pruritus numerical rating scale; DLQI, dermatological life quality index; POEM, patient-oriented eczema measure; ADCT, Atopic Dermatitis Control Tool; T, time-point in months

Time point	PROs correlated	ρs	p-value	Time point	PROs correlated	ρs	p-value
T0	ADCT - POEM	0,172	0,054	T24	ADCT - POEM	0,667	< 0,001
	ADCT - DLQI	0,316	< 0,001		ADCT - DLQI	0,710	< 0,001
	ADCT - P-NRS	0,155	0,084		ADCT - P-NRS	0,674	< 0,001
T1	ADCT - POEM	0,558	< 0,001	T28	ADCT - POEM	0,783	< 0,001
	ADCT - DLQI	0,440	< 0,001		ADCT - DLQI	0,760	< 0,001
	ADCT - P-NRS	0,465	< 0,001		ADCT - P-NRS	0,757	< 0,001
T4	ADCT - POEM	0,582	< 0,001	T32	ADCT - POEM	0,738	< 0,001
	ADCT - DLQI	0,613	< 0,001		ADCT - DLQI	0,795	< 0,001
	ADCT - P-NRS	0,404	< 0,001		ADCT - P-NRS	0,715	< 0,001
T8	ADCT - POEM	0,581	< 0,001	T36	ADCT - POEM	0,831	< 0,001
	ADCT - DLQI	0,574	< 0,001		ADCT - DLQI	0,812	< 0,001
	ADCT - P-NRS	0,454	< 0,001		ADCT - P-NRS	0,757	< 0,001
T12	ADCT - POEM	0,545	< 0,001	T40	ADCT - POEM	0,892	< 0,001
	ADCT - DLQI	0,483	< 0,001		ADCT - DLQI	0,788	< 0,001
	ADCT - P-NRS	0,429	< 0,001		ADCT - P-NRS	0,713	< 0,001
T16	ADCT - POEM	0,649	< 0,001	T44	ADCT - POEM	0,806	< 0,001
	ADCT - DLQI	0,545	< 0,001		ADCT - DLQI	0,768	< 0,001
	ADCT - P-NRS	0,594	< 0,001		ADCT - P-NRS	0,713	< 0,001
T20	ADCT - POEM	0,616	< 0,001	T48	ADCT - POEM	0,803	< 0,001
	ADCT - DLQI	0,597	< 0,001		ADCT - DLQI	0,757	< 0,001
	ADCT - P-NRS	0,629	< 0,001		ADCT - P-NRS	0,689	< 0,001

Table S2. Univariate regression and multivariate regression analyses for Numerical Rating Scale Pruritus. AD, atopic dermatitis, PN, prurigo nodularis; y/o, years old; OR, odd ratio; C.I., confidence interval.

	Univariate analysis			Multivariate analysis		
Covariates	OR	95% C.I.	p-value	OR	95% C.I.	p-value
**Baseline age** (over 60 y/o vs. under 60 y/o)	0,913	0,188-4,435	0,911	2,167	0,276-17,018	0,462
**AD onset** (late-onset vs. early-onset)	0,622	0,190-2,030	0,431	0,792	0,184-3,415	0,754
**Sex** (female vs. male)	0,926	0,333-2,573	0,882	1,088	0,373-3,173	0,877
**Atopic comorbidities**			0,979			0,948
1-2 comorbidities vs. 0 comorbidities	1,167	0,272-4,999	0,836	1,015	0,183-5,620	0,986
≥ 3 comorbidities vs. 0 comorbidities	1,1111	0,267-4,623	0,885	0,847	0,152-4,712	0,849
**Sensitization to a contact allergen** (yes vs. no)	1,000	0,301-3,321	1,0000	0,945	0,266-3,355	0,931
**AD phenotype**			0,141			0,412
Other than classic and PN vs. classic	1833	0,627-5,356	0,268	1,936	0,641-5,848	0,242
PN vs. classic	0,667	0,073-6,074	0,719	0,624	0,052-7,525	0,710
**Atopic family history** (yes vs. no)	2,118	0,763-5,883	0,150	2,209	0,705-6,915	0,174
**Intrinsic pattern vs. extrinsic pattern**	0,576	0,069-4,799	0,610	0,860	0,064-11,497	0,910

Table S3. Univariate regression and multivariate regression analyses for POEM. AD, atopic dermatitis, PN, prurigo nodularis, y/o, years old; POEM, patient-oriented eczema measure; OR, odd ratio; C.I., confidence interval

	Univariate analysis			Multivariate analysis		
Covariates	OR	95% C.I.	p-value	OR	95% C.I.	p-value
**Baseline age** (over 60 y/o vs. under 60 y/o)	1,655	0,478-5,732	0,427	2,699	0,510-14,278	0,243
**AD onset** (late-onset vs. early-onset)	1,510	0,595-3,834	0,386	1,638	0,482-5,568	0,429
**Sex** (female vs. male)	0,857	0,343-2,141	0,741	1,011	0,381-2,682	0,983
**Atopic comorbidities**			0,885			0,573
1-2 comorbidities vs. 0 comorbidities	1,284	0,355-4,639	0,703	2,538	0,448-14,397	0,293
≥ 3 comorbidities vs. 0 comorbidities	1,033	0,288-3,702	0,961	2,107	0,361-12,287	0,408
**Sensitization to a contact allergen** (yes vs. no)	0,904	0,304-2,686	0,856	0,665	0,206-2,144	0,495
**AD phenotype**			0,237			0,098
Other than classic and PN vs. classic	2,289	0,853-6,141	0,100	2,696	0,959-7,580	0,060
PN vs. classic	1,221	0,222-6,703	0,818	0,563	0,079-3,987	0,565
**Atopic family history** (yes vs. no)	1,030	0,422-2,515	0,948	1,406	0,502-3,938	0,516
**Intrinsic pattern vs. extrinsic pattern**	1,679	0,410-6,866	0,471	3,293	0,435-24,939	0,249

Table S4. Univariate regression and multivariate regression analyses for ADCT. AD, atopic dermatitis, PN, prurigo nodularis; y/o, years old; ADCT, atopic dermatitis control tool; OR, odd ratio; C.I., confidence interval.

	Univariate analysis			Multivariate analysis		
Covariates	OR	95% C.I.	p-value	OR	95% C.I.	p-value
**Baseline age** (over 60 y/o vs. under 60 y/o)	2,011	0,573-7,057	0,267	2,222	0,425-11,605	0,344
**AD onset** (late-onset vs. early-onset)	1,966	0,749-5,156	0,170	2,026	0,588-6,985	0,264
**Sex** (female vs. male)	0,887	0,339-2,324	0,808	1,090	0,393-3,025	0,869
**Atopic comorbidities**			0,519			0,740
1-2 comorbidities vs. 0 comorbidities	0,853	0,250-2,913	0,799	1,277	0,270-6,044	0,758
≥ 3 comorbidities vs. 0 comorbidities	0,514	0,145-1,828	0,304	0,821	0,162-4,163	0,812
**Sensitization to a contact allergen** (yes vs. no)	0,794	0,244-2,586	0,702	0,558	0,158-1,967	0,364
**AD phenotype**			0,575			0,255
Other than classic and PN vs. classic	1,714	0,621-4,735	0,298	2,026	0,700-5,864	0,193
PN vs. classic	1,221	0,222-6,703	0,818	0,511	0,072-3,632	0,502
**Atopic family history** (yes vs. no)	0,891	0,346-2,293	0,810	1,363	0,458-4,053	0,578
**Intrinsic pattern vs. extrinsic pattern**	2,021	0,489-8,352	0,331	1,954	0,282-13,531	0,497
